# A pilot study on pyroptosis related genes in peripheral blood mononuclear cells of non-small cell lung cancer patients

**DOI:** 10.1186/s12890-023-02456-x

**Published:** 2023-05-16

**Authors:** Ruoyu Song, Yongbin Wu, Shijun He, Wanxin Chen, Huan Chen, Qianlu Wang, Shouman Wang, Lan Xiao, Sichuang Tan, Sipin Tan

**Affiliations:** 1 Department of Pathophysiology, School of Basic Medicine Science, Central South University, Changsha, China; 2 Sepsis Translational Medicine Key Laboratory of Hunan Province, Central South University, Changsha, Hunan 410078 P.R. China; 3 National Medicine Functional Experimental Teaching Center, Central South University, Changsha, Hunan 410078 P.R. China; 4 Department of Laboratory Medicine, The Second Xiangya Hospital, Central South University, Changsha, Hunan 410011 People’s Republic of China; 5 Department of Thoracic Surgery, The Second Xiangya Hospital, Central South University, Changsha, Hunan 410011 People’s Republic of China; 6 Department of Traditional Chinese Medicine, The Third Xiangya Hospital, Central South University, Changsha, Hunan 410008 People’s Republic of China; 7 Department of Breast Surgery, Xiangya Hospital, Central South University, Changsha, Hunan 410008 People’s Republic of China

**Keywords:** Non-small cell lung cancer, Pyroptosis, Peripheral blood mononuclear cells, Early detection

## Abstract

**Objective:**

To investigate the GSDMD, CASP1, CASP4 and CASP5 expression in peripheral blood mononuclear cells of non-small cell lung cancer patients and analyze their clinical significance.

**Methods:**

71 non-small cell lung cancer patients were selected as the study group and 50 healthy individuals as the control group. The GSDMD, CASP1, CASP4 and CASP5 expression in peripheral blood mononuclear cells of the two groups were detected by real-time fluorescence quantitative PCR. The GSDMD, CASP1, CASP4, CASP5 expression and their relationship with the clinical characteristics of the patients were analyzed.

**Results:**

Compared with the control group, the GSDMD, CASP4 and CASP5 expression in PBMCs of lung cancer patients was significantly higher(P < 0.05). Lymph node metastasis had significant difference with the CASP4 and GSDMD expression (P < 0.05); tumor volume had significant difference with CASP1 and CASP5 expression (P < 0.05). The areas under predictive ROC curve of the GSDMD, CASP1, CASP4, and CASP5 mRNA expression were 0.629(P < 0.05), 0.574(p > 0.05), 0.701(P < 0.05) and 0.628(P < 0.05), the sensitivity values were 84.5%, 67.6% 43.7%, and 84.3%;the specificity values were 42%, 52%, 84% and 64%, respectively.

**Conclusion:**

GSDMD, CASP1, CASP4 and CASP5 gene expression are highly increased in PBMCs of non-small cell lung cancer patients and their expression are closely related to the clinical characteristics of patients. The early enhanced pyroptosis-related gene expression may be potential molecular markers for early diagnosis of non-small cell lung cancer.

**Supplementary Information:**

The online version contains supplementary material available at 10.1186/s12890-023-02456-x.

## Introduction

Lung cancer has a high incidence in the world and is one of the most malignant tumors. In 2020, there were more than 2 million new cases of lung cancer worldwide, and about 1.8 million people died of lung cancer [1]. Lung cancer is divided into small cell lung cancer (SCLC) and non-small cell lung cancer (NSCLC). NSCLC is the most common type of lung cancer, accounting for about 85% of all lung cancer cases [2].In the past few decades, targeted therapy and immunotherapy have become the focus of research on how to cure lung cancer. As a newly discovered form of programmed cell death, pyroptosis may provide a new idea for the treatment and diagnosis of lung cancer.

Pyroptosis is a mode of cell regulatory death accompanied by inflammatory response. In 2018, Nomenclature Committee on Cell Death (NCCD) defined it as regulated cell death with plasma membrane perforation formed by members of the Gasdermin protein family after inflammatory cysteinyl aspartate specific proteinase (caspase) activation [3]. According to the mechanism, pyroptosis can be divided into Caspase-1-dependent classical inflammatory body pathway and Caspase-4/-5-dependent non-classical inflammatory body pathway [4]. In the classical pathway of cell death, under the stimulation of pathogen-associated molecular patterns (PAMPs) and damage-associated molecular patterns (DAMPs), pattern recognition receptors (PRRs) such as NLR pyrin domain containing 3 (NLRP3), NLR containing a caspase recruitment domain 4 (NLRC4) and absent in melanoma 2 (AIM2) combine with the adapter protein ASC (apoptosis-associated speck-like protein containing a CARD) and Caspase-1 to form various inflammatory bodies to cleave GSDMD [5].The N-terminal of gasdermin-D (GSDMD) leads to the opening of cell membrane pores, which leads to pyroptosis [6, 7]. In the non-classical pathway of pyroptosis, lipopolysaccharides (LPS) directly binds to activate Caspase-4/5, and Caspase-4/5 induces pyroptosis by cutting GSDMD [8]. So, GSDMD and CASP1/4/5 play a key role in the occurrence of pyroptosis [9].

Peripheral blood mononuclear cells, including lymphocytes and monocytes, are important cellular components of the body’s immune response. The immune system can remove mutated cells from the body and early tumor cells. In patients with abnormal immune system or immunodeficiency, the incidence of tumor will be greatly increased, and the speed of canceration will be significantly accelerated [10]. PBMCs can secrete many kinds of cytokines, including interleukin and tumor necrosis factor (TNF). These different cytokines have more or less inhibitory effect on tumor. In recent years, it has been found that PBMCs play an important role in the occurrence and development of NSCLC [11].

In our pilot study in this paper, we investigated the expression profile of GSDMD, CASP1/4/5 in PBMCs of NSCLC patients, we further analyzed the relationship between these pyroptosis related genes and their clinical characteristics, so as to provide experimental basis for exploring pyroptosis as new forecasting molecules for NSCLC.

## Methods

### NSCLC patients and healthy controls

A total of 71 patients with NSCLC who underwent surgery in the second Xiangya hospital from December 2021 to February 2022 were enrolled, including 21 males and 11 females, aged from 41 to 70 years old; tumor diameter: > 5 cm 16 cases, ≤ 5 cm 16 cases; TNM staging: stage I + II in 9 cases, stage III + IV in 23 cases; lymph node metastasis occurred in 20 cases, no lymph node metastasis in 12 cases, distant metastasis in 11 cases and no distant metastasis in 21 cases. Inclusion criteria: [1] clinically diagnosed as NSCLC; [2] primary patients without any treatment before operation; [3] no other complications. 50 healthy subjects were selected in the control group. The study protocol was approved by the medical ethics committee of the Second Xiangya Hospital, Central South University. The ethical code was No.2020 Y557.

### Isolation of PBMCs and extraction of mRNA

Collect the blood samples collected by the patients within 24 h after admission from the laboratory department. PBMCs were isolated from the blood samples using density gradient centrifugation with Ficoll-Hypaque which purchased from 4 A Biotech Co., Ltd according to the manufacturer’s instruction. Total RNA was extracted from PBMCs, and genomic DNA (gDNA) was removed for reverse transcription. Trizol reagent and Evo M-MLV RT Mix Kit with gDNA clean were purchased from Accurate-Biology, Hunan, China.

### Reverse transcription and real-time PCR

RNA samples were reverse transcribed and 1ul cDNA from each sample was taken for RT-PCR detection. The reaction conditions were as follows: pre-denatured at 95 ℃, 50s, and 95 ℃, 10s, 60℃, 30s, 40 loops, and the relative expression of GSDMD and CASP1/4/5 was analyzed by 2^−ΔΔ^ CT method. GAPDH was used as the control gene for this experiment. The primers were purchased from Tsingke Biotechnology Co., Ltd. The primers used in PCR were listed as follows:

**Table:** 

Gene	NCBI reference sequence	primer sequence (5′-3′)
GSDMD	NM_001166237.1	F: GTGTGTCAACCTGTCTATCAAGG R: CATGGCATCGTAGAAGTGGAAG
CASP1	NM_001223.5	F: TTTCCGCAAGGTTCGATTTTCA R: GGCATCTGCGCTCTACCATC
CASP4	NM_001225.4	F: CAAGAGAAGCAACGTATGGCA R: AGGCAGATGGTCAAACTCTGTA
CASP5	NM_001136109.3	F: TCACCTGCCTGCAAGGAATG R: TCTTTTCGTCAACCACAGTGTAG

### Statistical analysis

GraphPadPrism5.0 was used for statistical analysis, and the measurement data were expressed by mean ± standard deviation. D’Agostino Pearson normality test was used to determine whether the data was normal distribution. For the data with normal distribution, F test was conducted to compare the variance, and then parametric T test was conducted for the data with the same variance and T test with Welch’s correction was conducted for the data with unequal variance; For data without normal distribution, Mann-Whitney test was conducted. The difference was considered to be statistically significant When the P-value < 0.05.

## Results

### GSDMD, CASP1/4/5 expression profile in PBMCs of NSCLC patients and healthy controls

**Fig. 1:**
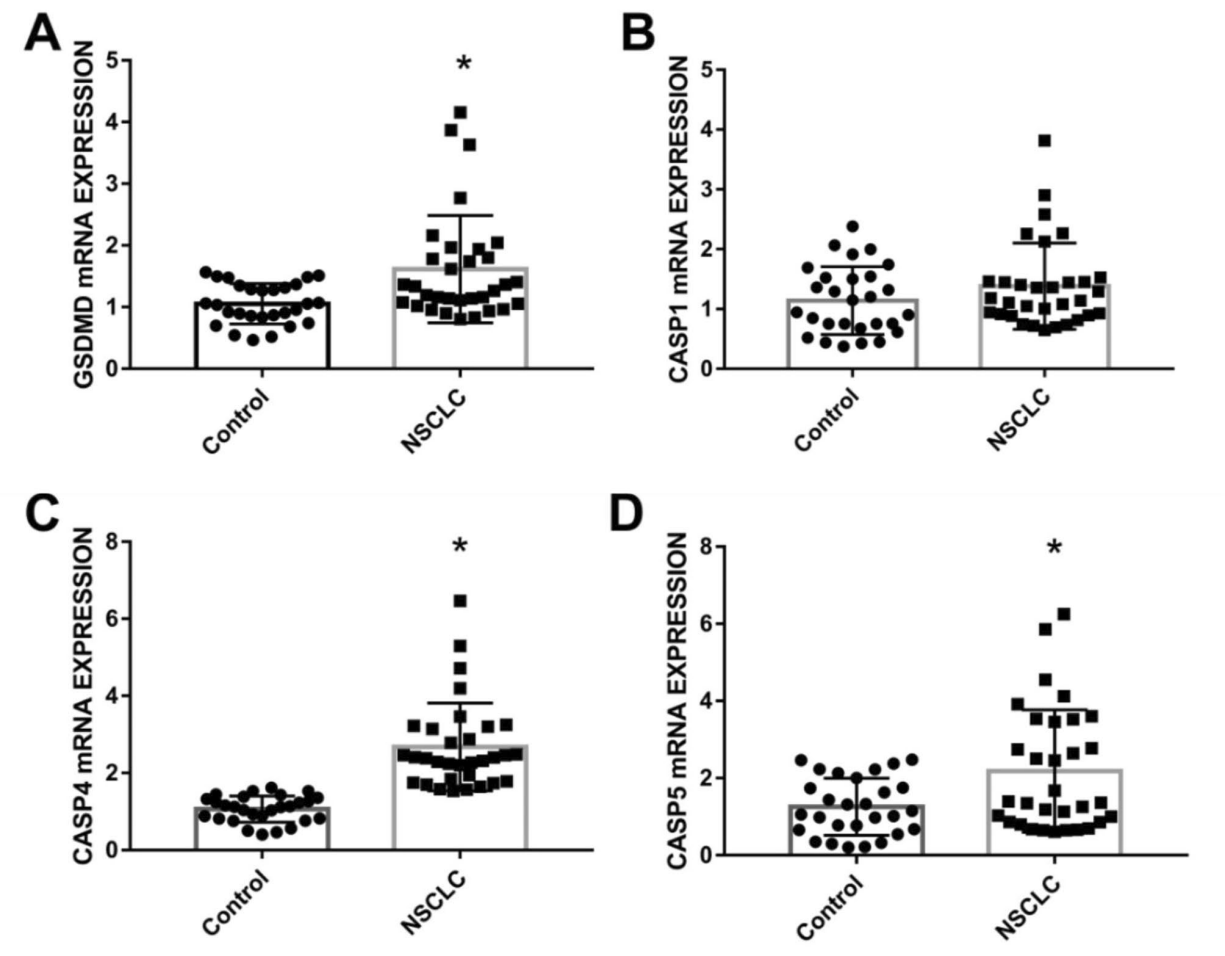
GSDMD(A), CASP1(B), CASP 4(C), CASP 5 expressions in PBMC of NSCLC patients and healthy controls

The GSDMD, CASP1/4/5 expressions differences in PBMCs of NSCLC patients and healthy controls were detected by real-time PCR. The results showed that both the GSDMD and CASP1/4/5 expressions in patients were higher than that of healthy controls, and the expressions of GSDMD, CASP4 and CASP5 were statistically higher than that of healthy controls(P < 0.05), as shown in Fig. 1 and supplementary Table 1.

### Relationship between GSDMD, CASP1/4/5 expressions and clinical characteristics of NSCLC patients

We further analyzed the relationship between the expression of GSDMD and CASP1/4/5 and the clinical characteristics of patients, as shown in Table 1. The expression of GSDMD in PBMCs was related to lymph node involvement(P < 0.05); the expression of CASP1 was related to tumor diameter(P < 0.05); the expression of CASP4 was related to TNM staging and lymph node involvement(P < 0.05); the expression of CASP5 in NSCLC patients was related to tumor diameter(P < 0.05). The GSDMD and CASP1/4/5 expressions in PBMCs were not related to age, gender, smoking history, pathological type and differentiation in patients with NSCLC(P > 0.05) (Supplementary Table 2).

**Table 1:** Relationship between expression of GSDMD and CASP1/4/5 and statistically significant clinical characteristics of patients

Clinical characteristics		n	GSDMD		Casp1		Casp4		Casp5	
			2^-ΔΔct^	P	2^-ΔΔct^	P	2^-ΔΔct^	P	2^-ΔΔct^	P
Tumor diameter	≤ 5 cm > 5 cm	36 35	1.283 ± 0.356 1.508 ± 0.267	0.361	0.836 ± 0.397 0.294 ± 0.175	**0.029**	1.692 ± 0.735 1.420 ± 0.816	0.620	1.209 ± 0.512 0.773 ± 0.401	**0.031**
Lymph node involvement	Yes No	40 31	1.730 ± 1.106 1.349 ± 0.976	**0.030**	0.842 ± 0.351 0.436 ± 0.238	0.764	1.934 ± 1.067 1.306 ± 1.052	**0.023**	1.356 ± 0.842 0.730 ± 0.514	0.327

### ROC curves of GSDMD and CASP1/4/5 expression in PBMCs

**Fig. 2:**
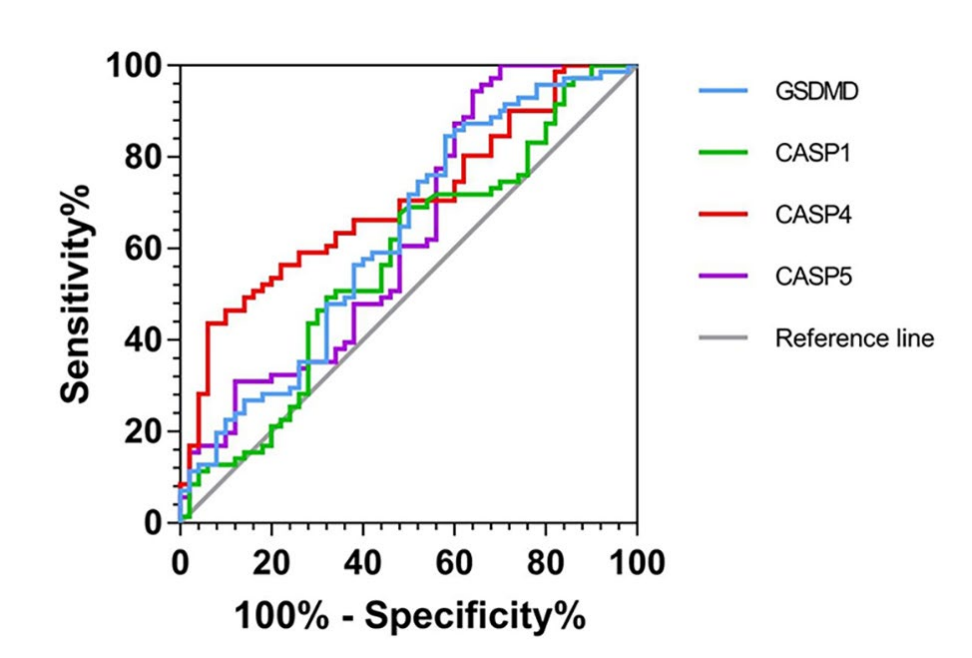
ROC curve of the GSDMD, CASP1, CASP4, and CASP5 mRNA expression

The areas under predictive ROC curve of the GSDMD, CASP1, CASP4, and CASP5 mRNA expression were 0.629(P < 0.05), 0.574(p > 0.05), 0.701(P < 0.05) and 0.628(P < 0.05), the sensitivity values were 84.5%, 67.6% 43.7%, and 84.3%;the specificity values were 42%, 52%, 84% and 64%, respectively, as shown in Supplementary Tables 3 and Fig. 2. It showed that the expression of pyroptosis related factors of PBMCs in NSCLC patients had certain value in diagnosing NSCLC.

## Discussion

The burden of incidence rate and case fatality rate of cancer increases rapidly all over the world. Functioning as the most common type of lung cancer, non-small cell lung cancer stays on top of various types of cancers. Patients suffered lung cancer generally have no typical symptoms in their early stage, and the 5-year survival rate of the patients is less than 15% [12]. At present, the main screening method of NSCLC is Computed Tomography (CT), but there are defects such as radiation exposure and high cost, and the high false positive results of the screening results could lead to overdiagnosis. Invasive techniques based on tissue biopsies could cause adverse reactions such as pneumothorax and hemorrhage. The limitations of tissue biopsy and tumor heterogeneity were also easy to produce false negative results. Therefore, it is very important to find early tumor markers of NSCLC and realize preoperative noninvasive early diagnosis.

As an inflammatory and programmed way of cell death, the molecular mechanism of pyroptosis has been gradually clarified with the deepening of research.

A large number of studies had shown that pyroptosis played an important role in the occurrence and development of NSCLC. In the development of NSCLC, pyroptosis also played a dual role, which could not only inhibit tumor cell proliferation, but also provide a suitable environment for tumor cell growth, thus promoting tumor cell proliferation. When inhibiting the expression of LncRNA-XIST oncogene, it could activate NLRP3 inflammatory bodies, increase the expression of caspase-1 and IL-1β, and then induce pyroptosis of tumor cells, ultimately inhibiting the occurrence and development of NSCLC. Knock-down of LncRNA-XIST inhibited NSCLC progression by triggering miR-335/SOD2/ROS signal pathway mediated pyroptotic cell death [13]. Thioredoxin interacting protein (TXNIP) could combine with IL-18 and IL-1β, initiate pyroptosis through overexpression of TXNIP and promote lung related chronic inflammatory response, while inhibiting the expression of TXNIP could reduce the formation of inflammatory bodies and inhibit lung related chronic inflammatory reactions [14]. The activation of NLRP3 inflammasome could promote the proliferation and migration of lung adenocarcinoma A549 cells by mediating the release of IL-18 and IL-1β through the caspase-1 dependent pyroptosis pathway, while blocking IL-18 and IL-1β signaling could inhibit the progression of lung cancer [15]. Therefore, pyroptosis related factors might have a dual mechanism of promoting and inhibiting the occurrence and development of NSCLC, but there were relatively few studies related to them at present, which could be used as a new research direction to further explore the dual role of pyroptosis in non-small cell lung cancer, and lay the foundation for seeking therapeutic targets.

More and more evidence showed that pyroptosis is related to the occurrence, development and therapeutic response of tumors [16, 17]. However, the vast majority studies have focused on whether pyroptosis occurs in tumor cells during tumorigenesis. It had been proved that GSDMD was highly expressed in NSCLC tumor tissue, and the level of expression was positively correlated with tumor size and TNM stage [14]. Xie et al. proved that the progression of NSCLC can be inhibited by activating the classical pyroptosis pathway in tumor cells [18]. But there were few previous studies on the pyroptosis of PBMCs in patients with NSCLC.

PBMC was an important part of the immune system which played an important role in anti-tumor immunity. The cells involved in anti-tumor immunity include NK cells, T cells, B cells, macrophages and dendritic cells (DC) [19]. At the same time, tumors could also inhibit the immune function of the body and induce immune tolerance to tumor cells through a variety of mechanisms, so as to escape the clearance of it by the immune system. PBMCs played an important role in the occurrence and development of tumor.

In this study, the expression levels of GSDMD and CASP1/4/5 in PBMCs of patients with NSCLC and healthy controls were detected by real-time PCR. It was found that the expression of GSDMD and CASP4/5 of patients with NSCLC was higher than that of healthy controls, and the difference was statistically significant(P < 0.05). The relationship between the expression of GSDMD and CASP1/4/5 and the clinical characteristics of patients was further analyzed. In our study, the expression of GSDMD was only related to lymph node metastasis(P < 0.05). Cancer progression is marked by dysfunctional tumor-infiltrating lymphocytes (TIL). It is highly possible that the inflammatory death of PBMC can cause the significant lymph node metastasis. The results showed that the expression of CASP1 in patients with tumor diameter > 5 cm was lower than that in patients with tumor diameter ≤ 5 cm(P < 0.05). The expression of CASP4 was related to lymph node metastasis (P < 0.05), and the expression of CASP5 was related to tumor size(P < 0.05).The mechanism of immune cell dysfunction in chronic infection during non-classical pyroptosis pathway activation was similar to that of anti-tumor immune cell dysfunction [20]. As the key molecules of non-classical pyroptosis pathway, the expression trend of CASP4 and CASP5 in PBMCs of patients with NSCLC was opposite to that of CASP1 in some clinical indicators. Although this difference did not show statistical significance, it also suggested that there may be a competitive relationship between the two ways of pyroptosis. Age, gender, smoking history, pathological type, degree of differentiation and distant metastasis of the tumor had no significant difference with the GSDMD, CASP1, CASP4 and CASP5 expression (P > 0.05).

The ROC results of this experiment showed that the detection of the expression of GSDMD and CASP1/4/5 in PBMCs had certain value in the diagnosis of NSCLC. The areas under predictive ROC curve of the GSDMD, CASP1, CASP4, and CASP5 mRNA expression were described previously. All these pyroptosis related factors in PBMCs had certain value in diagnosing NSCLC patients. GSDMD proved to be the most sensitive diagnostic molecules, however, its predictive value needs further verification.

Detection of pyroptosis related molecules may be helpful for the clinical diagnosis and prognosis evaluation of NSCLC. Currently, many studies had found that some drugs, such as conventional chemotherapy drugs, molecular targeted drugs, and immunotherapy drugs, could exert anti-tumor effects through pyroptosis, or could be used in combination with other pyroptosis promoters to enhance their drug sensitivity and reduce drug resistance, which was conducive to improving the efficacy of anticancer drugs, developing new drugs, and improving patient prognosis. In patients with NSCLC, researchers found that the GSDMD-N domain formed by GSDMD cleavage in CD8^+^ T cells may bind to the GSDMD-C domain. Once entering the target cell, GSDMD-N was liberated from GSDMD-C, bind to the target cell membrane, inducing target cell death to exert its role in killing tumor cells [21]. Wang had found that caspase-1 dependent pyroptosis induced by the lipid-lowering drug simvastatin occurred in lung cancer cells, indicating that simvastatin might be a potential drug for clinical treatment of NSCLC [15]. Further in-depth research on key molecules such as caspase and GSDMD in pyroptosis and their regulatory molecular mechanisms was of great significance in the diagnosis and treatment of NSCLC. The exploration of pyroptosis and its molecular mechanism might provide new therapeutic strategies and targets for cancer patients, with broad research prospects.

This study also had some limitations. First of all, the number of cases included in our experimental group and control group was relatively small, and our data were only collected in a single institution. Therefore, we carefully examined the clinical information of patients before collecting patient samples to ensure that the patients included in the experimental study met the standards. Secondly, we evaluated the expression of pyroptosis-related molecules in PBMCs of patients with NSCLC at only one time point, and did not follow up to evaluate the changes of the expression of these molecules with time. Different treatment methods might also affect the expression of pyroptosis related genes. In addition, although some results, including ROC curve analysis, have reference significance, the credibility of the experimental results still need to be further proved, which may also be related to the insufficient number of samples, sample differences or case selection. In this experiment, we collected clinical samples and attempted to identify the relationship between the expression level of pyroptosis related genes and clinical characteristics, without involving research on the regulatory pathway of pyroptosis. Therefore, in the future, we should expand the number of cases in the experimental study and continue to further investigate the expression and mechanism of pyroptosis of PBMCs in NSCLC.Consisting of monocytes, T cells, B cells and natural killer cells, PBMCs could be considered the first line of defense of the immune system against cancer. The change of activity and function of PBMCs was a key factor in tumor immune escape [22, 23]. Once tumor immune escape occurred, the expression of some molecules in PBMCs would be different, and this change might be earlier than that of precancerous lesions. When pyroptosis occurred in PBMCs, it changed the state of the patient’s immune system, and the internal environment would be disordered with the occurrence and development of the tumor. The occurrence of tumor will affect the metabolism of immune cells [24], which might explain why the expression of pyroptosis molecules was related to the stage of tumor development. Pyroptosis occurred in tumor was beneficial to cure cancer, but if pyroptosis occurred in PBMCs, it would inevitably affect the immune state of the body, which was adverse for the body [17, 25, 26]. The differential expression of PBMCs molecules did not depend on the substantial tumor load, and their expression was involved in the immune system’s recognition of cancer and the process of tumor cells escaping from the immune system. Moreover, the immune escape of cancer and tumor cells recognized by the immune system occurred in the early stage of tumor, so the differential expression of pyroptosis-related molecules could reflect the state of early tumor occurrence [27]. PBMCs could produce a large number of high-quality RNA, and gene expression could be measured reliably by real-time fluorescence quantitative PCR. Therefore, the expression of pyroptosis-related molecules in PBMCs as molecular markers might have higher diagnostic value in theory.

## Conclusion

In summary, pyroptosis-related molecules GSDMD and CASP1/4/5 were highly expressed in PBMCs of patients with NSCLC, and their expression levels were closely related to the clinical features of patients, and might be potential molecular markers for early diagnosis and evaluation the severity of this disease. Pyroptosis can provide a new idea for early detection and treatment of lung cancer.

## Electronic supplementary material

Below is the link to the electronic supplementary material.

Supplementary Material 1

Supplementary Material 2

Supplementary Material 3

## Data Availability

The datasets used and analyzed during the current study are not publicly available due to the experimental data of this study involves the privacy of patients but can be obtained from the corresponding authors according to reasonable requirements.
