# Supplementary material for: A pilot study on pyroptosis related genes in peripheral blood mononuclear cells of non-small cell lung cancer patients
Source: BMC Pulm Med. 2023 May 16;23:174. doi: 10.1186/s12890-023-02456-x (PMC10190026; doi:10.1186/s12890-023-02456-x)
Supplement: Supplementary file 1 — Supplementary Material 1 [file 12890_2023_2456_MOESM1_ESM.docx]

|  | Control(n=50) | NSCLC(n=71) | P |
| --- | --- | --- | --- |
|  | 2^-ΔΔct^ | |  |
| GSDMD | 1.029±0.798 | 1.395±0.945 | 0.017 |
| CASP1 | 0.491±0.398 | 0.578±0.425 | 0.251 |
| CASP4 | 0.795±0.628 | 1.502±0.954 | 0.000 |
| CASP5 | 0.676±0.605 | 0.957±0.774 | 0.011 |

**Supplementary Table 1** Detection of GSDMD and CASP1/4/5 expression in PBMCs by RT-PCR
