# Supplementary material for: A pilot study on pyroptosis related genes in peripheral blood mononuclear cells of non-small cell lung cancer patients
Source: BMC Pulm Med. 2023 May 16;23:174. doi: 10.1186/s12890-023-02456-x (PMC10190026; doi:10.1186/s12890-023-02456-x)
Supplement: Supplementary file 2 — Supplementary Material 2 [file 12890_2023_2456_MOESM2_ESM.docx]

|  | n | GSDMD | | CASP1 | | CASP4 | | CASP5 | |
| --- | --- | --- | --- | --- | --- | --- | --- | --- | --- |
|  |  | AUC | P | AUC | P | AUC | P | AUC | P |
| Control | 50 | 0.629 | 0.016 | 0.574 | 0.164 | 0.701 | 0.000 | 0.628 | 0.017 |
| NSCLC | 71 |  |  |  |  |  |  |  |  |

**Supplementary Table3 ROC curves of GSDMD and CASP1/4/5 expression in PBMCs**
