# Supplementary material for: A pilot study on pyroptosis related genes in peripheral blood mononuclear cells of non-small cell lung cancer patients
Source: BMC Pulm Med. 2023 May 16;23:174. doi: 10.1186/s12890-023-02456-x (PMC10190026; doi:10.1186/s12890-023-02456-x)
Supplement: Supplementary file 3 — Supplementary Material 3 [file 12890_2023_2456_MOESM3_ESM.docx]

| Clinical characteristics | | n | GSDMD | | Casp1 | | Casp4 | | Casp5 | |
| --- | --- | --- | --- | --- | --- | --- | --- | --- | --- | --- |
|  |  |  | 2^-ΔΔct^ | P | 2^-ΔΔct^ | P | 2^-ΔΔct^ | P | 2^-ΔΔct^ | P |
| Age | <60  ≥60 | 35  36 | 1.452±0.836  1.430±0.941 | 0.852 | 0.752±0.419  0.521±0.286 | 0.691 | 1.649±1.230  1.430±0.841 | 0.227 | 1.165±1.010  0.834±0.677 | 0.263 |
| Gender | Male  Female | 49  22 | 0.934± 0.670  1.661± 1.238 | 0.061 | 0.649± 0.220  0.670± 0.315 | 1.038 | 1.607± 0.830  1.503± 1.012 | 0.165 | 0.940± 0.638  1.347± 0.941 | 0.764 |
| Smoking history | Yes  No | 38  33 | 1.391±0.739  1.579±0.873 | 0.462 | 0.634±0.360  0.611±0.318 | 0.706 | 1.904±0.760  1.230±1.020 | 0.839 | 1.648±1.206  0.864±0.528 | 0.730 |
| Pathological type | Adenocarcinoma  Squamous cell carcinoma | 42  29 | 1.673±1.164  1.221±0.594 | 0.264 | 0.416±0.259  0.942±0.407 | 0.130 | 1.519±1.127  1.673±1.193 | 0.721 | 0.760±0.320  1.943±1.235 | 0.781 |
| Differentiation | Poor  Well | 31  40 | 1.037±0.530  1.591±1.195 | 0.101 | 0.580±0.304  0.699±0.435 | 0.705 | 1.642±0.658  1.488±0.901 | 0.255 | 1.613±0.856  0.509±0.307 | 0.380 |
| Metastasis | Yes  No | 29  42 | 1.762±1.462  1.284±0.730 | 0.307 | 0.431±0.198  0.865±0.523 | 0.068 | 1.493±1.043  1.562±0.885 | 0.098 | 1.346±0.458  0.852±0.269 | 0.510 |
| TNM | Ⅰ+Ⅱ  Ⅲ+Ⅳ | 31  40 | 1.596±1.374  1.307±0.826 | 0.406 | 0.561±0.236  0.743±0.347 | 0.064 | 1.476±1.130  1.558±1.208 | 0.116 | 1.375±0.940  0.815±0.647 | 0.493 |

**Supplementary Table 2. Relationship between expression of GSDMD and CASP1/4/5 and statistically non-significant clinical characteristics of patients**
